# Rural Home Annotation Dataset Mapped by Citizen Scientists in Satellite Imagery

**DOI:** 10.1016/j.dib.2022.108262

**Published:** 2022-05-11

**Authors:** Alycia Leonard, Scot Wheeler, Malcolm McCulloch

**Affiliations:** Department of Engineering Science, University of Oxford, Parks Road, Oxford, OX1 3PJ, UK

**Keywords:** Citizen science, Remote sensing, Geographic information systems, Online participation, Satellite mapping, Computer vision, Object detection

## Abstract

This article presents a geolocated dataset of rural home annotations on very high resolution satellite imagery from Uganda, Kenya, and Sierra Leone. This dataset was produced through a citizen science project called “Power to the People”, which mapped rural homes for electrical infrastructure planning and computer-vision-based mapping. Additional details on this work are presented in “Power to the People: Applying citizen science to home-level mapping for rural energy access” [1]. 578,010 home annotations were made on approximately 1,267 km^2^ of imagery over 179 days by over 6,000 volunteers. The bounding-box annotations produced in this work have been anonymized and georeferenced. These raw annotations were found to have a precision of 49% and recall of 93% compared to a researcher-generated set of gold standard annotations. Data on roof colour and shape were also collected and are provided. Metadata about the sensors used to capture the original images and the annotation process are also attached to data records. While this dataset was collected for electrical infrastructure planning research, it can be useful in diverse sectors, including humanitarian assistance and public health.

## Specifications Table

**Table:** 

Subject	Geographical Information System
Specific subject area	Remote mapping accomplished through citizen science using satellite imagery. This can be used as training data in computer vision mapping for renewable energy infrastructure planning among other applications.
Type of data	Data table (CSV)
How the data were acquired	Online citizen science. Bounding box annotations were collected on very high resolution (VHR) satellite imagery through the “Power to the People” (PTTP) citizen science project hosted on the Zooniverse platform. Imagery was captured by the Superview-1 constellation, the Disaster Monitoring Constellation 3 (DMC-3), and the Korea Multi-Purpose Satellite (KOMPSAT) 3A.
Data format	Raw annotation CSV exported from the Zooniverse. Minor post-processing was undertaken for georeferencing and ease of interpretation, but no changes were made to the underlying data.
Description of data collection	Homes were annotated on satellite imagery samples in rural Kenya, Uganda, and Sierra Leone by citizen scientists on the Zooniverse platform. These countries were selected given their ongoing rural electrification efforts at different stages, home location data gaps in OSM, and diverse rural home styles (e.g. agricultural, clustered community, refugee, etc.). The ground sample distance of all images was ≤ 1 m. The citizen science platform was open to any global contributors with an internet connection.
Data source location	Countries: Kenya, Uganda, and Sierra Leone. Coordinates for collected data are included in dataset.
Data accessibility	Repository name: Mendeley Data, DOI: 10.17632/xw6gr8p2cn.1, Direct URL to data: www.doi.org/10.17632/xw6gr8p2cn.1.
Related research article	A. Leonard, S. Wheeler, M. McCulloch, Power to the people: Applying citizen science and computer vision to home mapping for rural energy access, International Journal of Applied Earth Observation and Geoinformation 108 (2022) 102748.

## Value of the Data

• Rural homes are typically underrepresented in available global population and home location datasets. These data help to fill that gap.
• State-of-art vector datasets (i.e. OpenStreetMap) tend to miss certain rural housing styles prevalent in developing countries (e.g. thatched roof homesteads). They can also have large data gaps in these areas. The data presented here target under-mapped areas and provide examples of the rural housing styles which are frequently missed.
• State-of-the-art raster datasets (i.e. Gridded Population of the World, High-Resolution Settlement Layer) can also have accuracy issues and can misrepresent small communities in applications that require home-level location data such as electrical grid design. These data usefully provide data at home-level resolution.
• These data can be used directly in GIS for rural infrastructure planning. Mapping rural populations in detail has broad applications in sciences, engineering, and humanities.
• These data can also be used as training data for computer-vision-driven mapping. Automated home mapping algorithms can be trained using this data to better locate rural housing styles quickly and at scale.

## 1 Data Description

The data associated with this work are contained in two CSV files:

• *pttp_annotations*, which presents each home annotation (pixel-wise and in geographic coordinates) in a separate row.
• *pttp_subjects*, which presents details and metadata about each subject in a separate row.

These data can be easily linked as needed. For instance, to append subject data to each annotation, one could join the data tables on the *subject_id* field included in both. The fields included in these data tables are defined as follows:

**pttp_annotations**

• *subject_id*: Unique number identifying the subject containing the annotation. This can be used to join the information from *pttp_subjects*.
• *classification_id*: Unique number identifying the classification containing the annotation. One classification represents one set of annotations on one subject by one contributor.
• *user_id*: Unique number anonymously identifying the contributor who made the annotation.
• *x, y*: Corner coordinate of the annotation in pixels originally provided in the Zooniverse data export.
• *width, height*: Width and height of the annotation in pixels originally provided in the Zooniverse export.
• *angle*: Angle of the annotation in degrees (clockwise from the positive x axis) originally provided in the Zooniverse export.
• *x_center, y_center*: Center point of the annotation box in pixels.
• $x_{n},y_{n}$ : Corner coordinates of annotation box in pixels.
• $x_{n}\_\mathrm{coord},y_{n}\_\mathrm{coord}$ : Corner coordinates of the annotation box in geographic coordinates. These are provided in the original referencing system of the annotated satellite images, which are WGS 84 in the UTM zone appropriate to the location in question (accessible in subject metadata).
• *roof_colour, roof_shape*: Colour and shape of the roof indicated by this annotation. For roof_colour: 0 = white or light-coloured, 1 = brown, 2 = other. For roof_shape: 0 = square or rectangular, 1 = circular or rounded, 2 = other.

**pttp_subjects**

• *subject_id*: Unique number identifying each subject.
• *workflow_id*: Unique number identifying the workflow containing the subject.
• *subject_set_id*: Unique number identifying the subject set (i.e. original satellite image) containing the subject.
• *metadata*: Information about the subject uploaded to the Zooniverse platform, including a general description of area, the satellite which captured the images, and filenames.
• *locations*: Web links to individual image tiles constituting this subject.
• *classifications_count*: How many times the subject was classified (i.e. viewed and annotated by one user).
• *retired_at*: Date the subject was retired from the Zooniverse platform.
• *retirement_reason*: Reason why the subject was retired from the Zooniverse platform.
• *created_at*: Date the subject was created on the Zooniverse platform.
• *T0_yes_count, T0_no_count, T0_None_count*: Number of times the answers “yes” and “no” were provided to question T0 (i.e. whether the user sees any homes in the image). “None” indicates no answer.
• *T2_yes_count, T2_there-were-too-many-to-label-them-all_count, T2_None_count*: Number of times the answers “yes” and “There were too many to label them all” were provided to question T2 (i.e. whether the user had annotated all homes they could see in the image). “None” indicates no answer.
• *tile_min_x, tile_min_y, tile_max_x, tile_max_y*: Minimum and maximum geographic corner coordinates of the image tiles for this subject.

## 2 Experimental Design, Materials and Methods

This work uses citizen science to annotate home locations on high-resolution satellite imagery. This approach has proven popular and effective in numerous biology and ecology projects, such as Penguin Watch [2], Snapshot Serengheti [3], and iNaturalist [4], to facilitate tracking and conservation efforts and generate annotated training datasets. Here, the same principles are applied with satellite imagery to map rural homes which are underrepresented in available datasets, enabling GIS analysis and computer vision driven mapping.

### 2.1 Data preparation

**Fig. 1:**
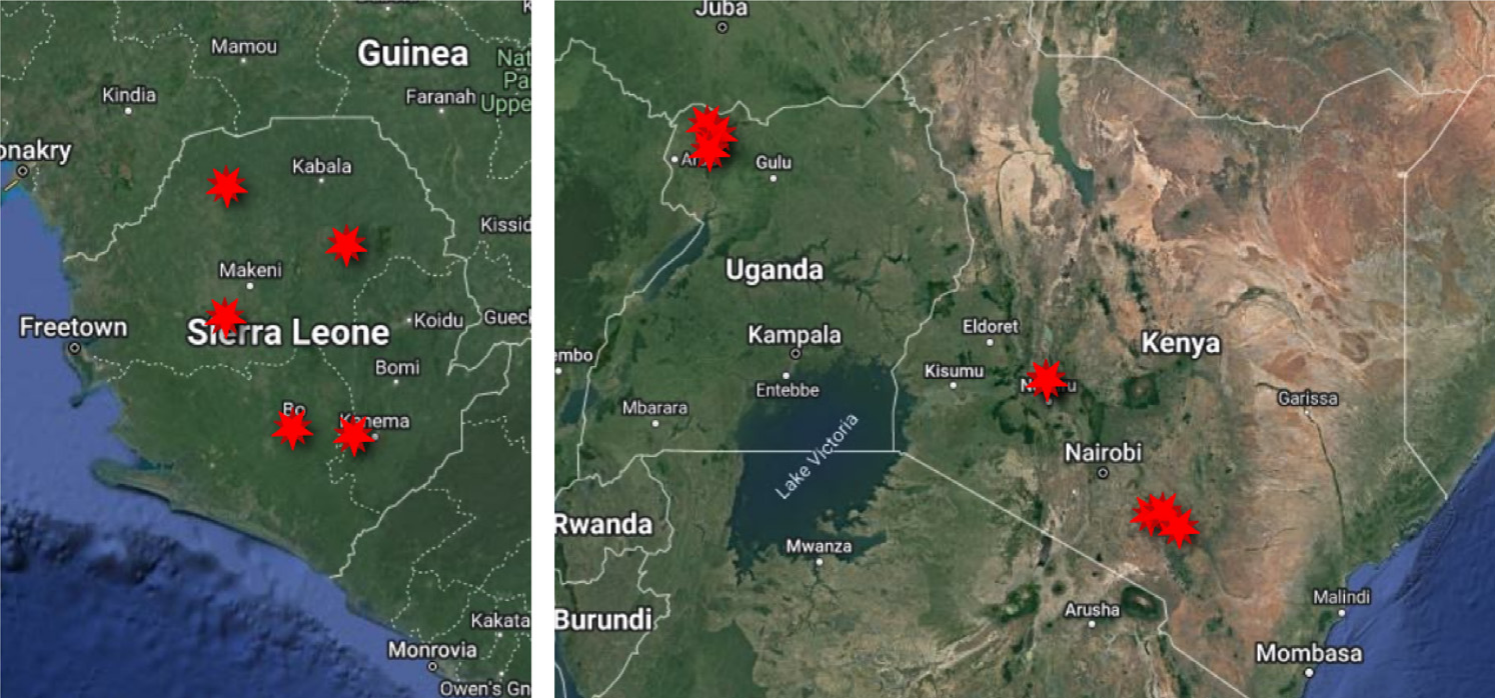
Locations of satellite imagery samples mapped at home level in the PTTP citizen science project are indicated by red stars.

Satellite images used in the Power to the People (PTTP) citizen science project were captured by the Superview-1 constellation (also known as Gaojing-1), DMC-3 (also known as TripleSat), and KOMPSAT 3A. Each of these has a panchromatic ground sample distance ≤ 1 m or lower to enable accurate home-level annotation. Images from rural Kenya, Uganda, and Sierra Leone were annotated. These countries were selected given their ongoing rural electrification efforts at different stages, home location data gaps in OSM, and diverse rural settlement styles (e.g. agricultural, clustered community, refugee, etc.). Specific imagery samples were selected to include rural housing styles which are underrepresented in home level maps and computer vision training data (e.g. thatched roofs, multi-structure homesteads, homes far from roadways or surrounded by vegetation, refugee homes). Sampled areas are shown in Fig. 1.

Satellite images were pre-processed with pansharpening and histogram correction for higher resolution and natural colour appearance respectively. They were then tiled and up-sampled into uniform 512× 512 squares. Georeferencing information and metadata from each tile was exported and saved separately. These images were uploaded to the Zooniverse platform as three-tile “subjects”. Each subject included three images of the same area, composed of multispectral imagery visualized in RGB, panchromatic imagery visualized in greyscale, and pansharpened imagery. Pansharpened imagery was shown by default, but contributors could cycle through others with on-screen controls.

### 2.2 Image annotation

**Fig. 2:**
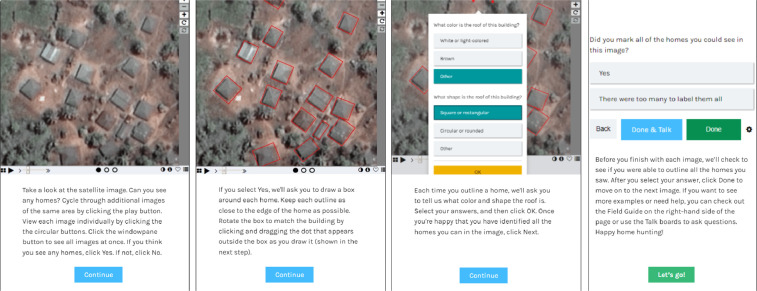
Tutorial on annotation steps provided to new citizen scientists on PTTP, proceeding from first (left) to right (last) instruction screen.

The Zooniverse online citizen science platform was used to collect imagery annotations. Zooniverse is the largest and most popular platform for online citizen science, with an existing base of nearly 2.5 million volunteer contributors across all projects [5]. On the Zooniverse, PTTP had its own homepage which linking to its annotation workflows, informational pages, and discussion boards^1^. Three parallel annotation workflows were created for the studied countries, each of which had the same structure and instructions. Prior to starting annotation, each volunteer was shown a walk-through tutorial explaining the annotation steps, as shown in Fig. 2. They also had access to a “Field guide” demonstrating different annotation contexts, and could contact other volunteers or researchers via a “Talk” discussion forum for additional assistance if needed.

In the PTTP annotation workflow, the contributor was first asked whether they could see any homes in a presented subject. If they did not, they were presented with a new subject. If they did, they were asked to draw a box around any homes or buildings they saw. They were also asked to identify the color and shape of each roof, and had the ability to rotate the boxes they drew to align with roofs as accurately as possible. Once they had annotated as many homes as possible, they were asked confirm whether they had annotated all the homes they could see, and to confirm their annotations before moving to another subject. Each subject was classified (i.e. viewed and annotated in the above-described workflow) by multiple contributors. If the first five contributors to classify a subject all indicated that there were no homes, the subject was retired. If any of the first five did see any homes, the subject was classified by ten contributors before retirement.

**Fig. 3:**
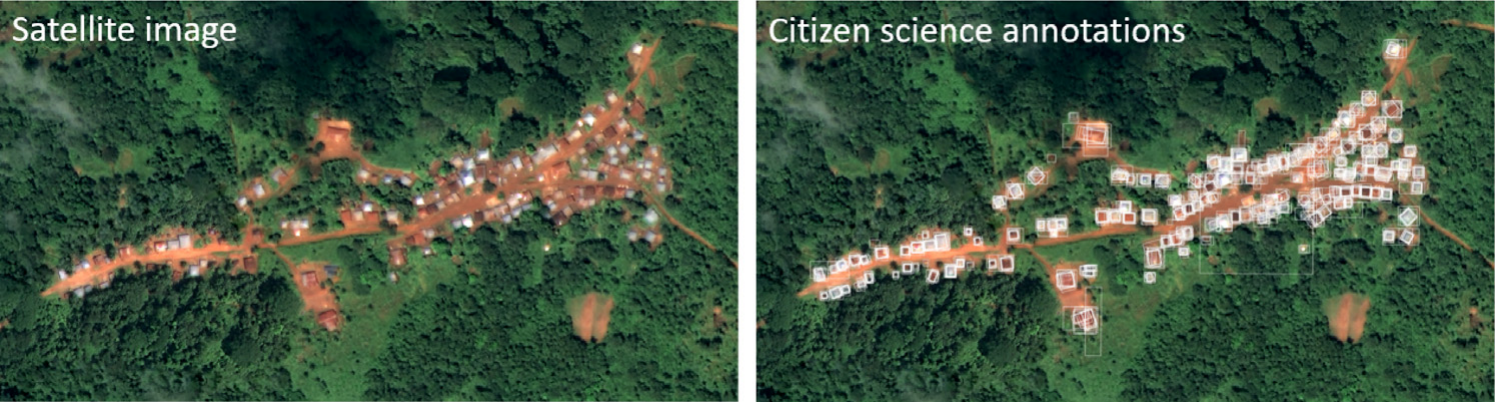
Example of the data produced through PTTP citizen science project for a community in Sierra Leone.

PTTP ran from 2nd March to 28th August 2020, during which time 578,010 annotations were made by over 6,000 volunteers on 74,802 subjects. An example of these annotations is shown for a small area in Sierra Leone in Fig. 3. Through a post-project evaluation survey, it was found that the project volunteer base represented at least six continents and 25 countries, though project web analytics indicated traffic from many more countries than were represented in the survey. A large number of volunteers on PTTP were from the United States or United Kingdom, as these countries have large existing contributor bases on the Zooniverse platform. Volunteers spanned diverse ages, occupations, and education levels, and there were more women than men represented. Note that the composition and motivations of the citizen science community are studied in detail in [6]. Though the project was promoted through social media as well as by various universities and large events, the majority of the volunteer base found PTTP and decided to join via the Zooniverse website.

### 2.3 Validation

To examine the accuracy of data, classifications on a random sample of 188 subjects made by author AL were employed as a “gold standard” (GS). This was used to calculate precision (P), recall (R), and $F_{1}$ score (i.e. harmonic mean of P and R) for the CS annotations, defined as:

(1) $$P=\frac{T_{P}}{T_{P}+F_{P}}$$

(2) $$R=\frac{T_{P}}{T_{P}+F_{N}}$$

(3) $$F_{1}=2\frac{P\times R}{P+R}$$

where $T_{P}$ represents true positives (i.e. positive predictions which agree with ground truth), $F_{P}$ represents false positives (i.e. positive predictions which disagree with ground truth), and $F_{N}$ represents false negatives (i.e. negative predictions which disagree with ground truth). A threshold intersection over union (IoU) value was necessary to differentiate between true and false positives. (IoU) can be intuitively understood here as an amount of overlap needed between a CS and GS annotation to classify that annotation as correct. IoU can be defined between any A and B as:

(4) $$IoU=\frac{A\cap B}{A\cup B}$$

An IoU threshold of 0.5 was used, so IoU≥0.5 between a citizen science annotation and any GS annotation was counted as $T_{P}$, IoU<0.5 between a citizen science annotation and any GS annotation was counted as a $F_{P}$, and IoU<0.5 between a GS annotation and any citizen science annotation was counted as a $F_{N}$.

Results were P=0.492, R=0.933, and $F_{1}=0.644$. The high R value may indicate that citizen scientists found most homes, but is also influenced by the fact that multiple overlapping annotations each count as a distinct $T_{P}$. The lower P value indicates a substantial amount of $F_{P}$ amongst the annotations.

Annotations were experimentally clustered using HDBSCAN* to see whether accuracy could be improved. HDBSCAN* was run with $m_{clSize}$ values from 2 to 10. The highest P (0.689) was seen for $m_{clSize}=4$ while the highest R (0.487) and $F_{1}$ (0.568) were seen at $m_{clSize}=2$. While these clustered annotations are not included in the data repository, they can be re-created as needed. Refer to the ”Code availability” for appropriate tools to do so.

### 2.4 Data post-processing

The annotation records were translated to a set of four corner coordinates. The five data points recorded in Zooniverse exports for the position of a rotated bounding box are: an x and y coordinate in pixels, an angle (hereafter θ) of rotation, and the width (w) and height (h) of the box. While the Zooniverse is generally highly documented, the Zooniverse documentation was ambiguous about the exact position of the x and y coordinate provided (i.e. whether this point represents the center of the box or any of the corners), and whether θ is in radians or degrees. Through data visualisation and cross-checking with satellite imagery and GS annotations, it was determined that the x and y coordinate represents the top right corner of the bounding box, measured from the top right corner of the image, and that θ is measured from 0 in degrees *in a negative (i.e. clockwise) direction* about the center of the defined rectangle. This is likely due to the treatment of the upper right corner of an image as (0,0) in most image processing applications. Given this, one could consider the picture in a vertically flipped state, which would make the bottom left corner the conventional (0,0) for the Cartesian plane and which would make θ actually point in the conventionally positive counter-clockwise direction. However, in this case, the images were not flipped, and instead a −θ was used when converting annotation records to corner coordinates. In other words, to get the $x_{n}$ and $y_{n}$ coordinates for each corner of the rotated bounding box for n=1:4, the “unrotated” bounding box were first generated by adding w and h to x and y respectively; then, all points were rotated about the center point of this box (i.e. $x_{c}$, $y_{c}$) by −θ. The “unrotated” annotation corners ($x_{n_{u}}$, $y_{n_{u}}$) were first calculated as:

(5) $$\left(x_{1_{u}},y_{1_{u}}\right)=\left(x,y\right)$$

(6) $$\left(x_{2_{u}},y_{2_{u}}\right)=\left(x+w,y\right)$$

(7) $$\left(x_{3_{u}},y_{3_{u}}\right)=\left(x+w,y+h\right)$$

(8) $$\left(x_{4_{u}},y_{4_{u}}\right)=\left(x,y+h\right)$$

Then, the box center was obtained as:

(9) $$\left(x_{c},y_{c}\right)=\left(x+\frac{w}{2},y+\frac{h}{2}\right)$$

Finally, rotated annotation corner coordinates were obtained for each corner n as:

(10) $$x_{n}=x_{c}+\left(x_{n_{u}}-x_{c}\right)\cos\left(-\theta \right)+\left(y_{n_{u}}-y_{c}\right)\sin\left(-\theta \right)$$

(11) $$y_{n}=y_{c}-\left(x_{n_{u}}-x_{c}\right)\sin\left(-\theta \right)+\left(y_{n_{u}}-y_{c}\right)\cos\left(-\theta \right)$$

### 2.5 Code availability

Python bindings of GDAL (https://gdal.org/api/python.html) were used for pansharpening and tiling. QGIS (https://qgis.org/en/site/) was used to colour-correct image histograms. ImageMagick Mogrify (https://imagemagick.org/script/mogrify.php) was used to upsample image tiles and convert TIF image tiles to PNG image tiles. The Zooniverse project builder (https://www.zooniverse.org/lab) was used to construct and manage the online citizen science platform. The Panoptes Zooniverse API was used to upload subjects to the online platform (https://panoptes.docs.apiary.io/#). Annotation data was also downloaded from the Zooniverse project builder, and was “flattened” (i.e. JSON chunks within the export CSV were broken down into CSV columns) using the panoptes_aggregation Python package (https://aggregation-caesar.zooniverse.org/Scripts.html). This package was also used for experimental HDBSCAN* clustering of data. Customs scripts to (1) generate a CSV with one annotation per row instead of one classification per row (i.e. the result of the panoptes_aggregation flattening); (2) append georeferencing information to subjects and annotations; (3) count T0 and T2 responses to append to subjects; and (4) translate from (x, y, width, height, angle) to corner coordinates ($x_{n},y_{n}$) for n=1:4; were written by author AL. The roof_colour and roof_shape columns were unpacked from the original “details” column using the Excel “Text to columns” function and subsequently deleting extraneous brackets.

## Ethics Statements

All data is presented anonymously. Citizen scientists cannot be identified and provided no personal information when annotating homes on PTTP. Open sharing of data wherever possible adheres to the principles of citizen science [7].

## CRediT authorship contribution statement

**Alycia Leonard:** Conceptualization, Methodology, Software, Validation, Formal analysis, Data curation, Writing – original draft, Writing – review & editing, Visualization, Funding acquisition, Project administration. **Scot Wheeler:** Conceptualization, Writing – review & editing, Visualization. **Malcolm McCulloch:** Conceptualization, Methodology, Writing – review & editing, Supervision.

## Declaration of Competing Interest

The authors declare that they have no known competing financial interests or personal relationships that could have appeared to influence the work reported in this paper.

## Data Availability

Rural Home Annotation Dataset Mapped by Citizen Scientists in Satellite Imagery (Original data) (Research Data).

## Footnotes

1 See: https://www.zooniverse.org/projects/alycialeonard/power-to-the-people
